# The Magical Combination of Polymer Science and Fluorometry

**DOI:** 10.3390/polym12040876

**Published:** 2020-04-10

**Authors:** Seiichi Uchiyama

**Affiliations:** Graduate School of Pharmaceutical Sciences, The University of Tokyo, Tokyo 113-0033, Japan; seiichi@mol.f.u-tokyo.ac.jp; Tel.: +81-3-5841-4768

I am very pleased to announce the publication of “Fluorescent Polymers for Sensing and Imaging”. This Special Issue includes a review, thirteen articles, and one communication, which represent the contributions of seventy researchers in nine countries. When I received an invitation to serve as a guest editor from the editorial office of Polymers, I immediately recalled the first time I read the striking results published in 2000 by Swager et al., who reported a dramatic increase in pH sensitivity due to the amplifying effects of polymers [1]. Before obtaining my PhD in 2002, my research was focused on developing novel fluorescent sensing systems using only small organic molecules. The Swager paper provided fresh insight into polymeric architecture, which often afforded extremely high sensitivity that could not be achieved with small molecules. At the same time, a fluorescent polymeric thermometer with sub-degree temperature resolution was developed in my laboratory [2], which, amazingly, enabled intracellular thermometry [3]. Of course, the robustness and multiple functionality of a polymer motif are different advantages.

I have made two contributions to the Special Issue as a guest editor. In my review article [4], I summarize the findings of a long-term investigation performed in collaboration with Prof. de Silva on polymer-based sensing systems and polymer-specific microenvironments. In a research article [5], I present new intracellular thermometry results obtained using a cationic fluorescent nanogel thermometer. Measuring the temperature within living cells with polymeric sensing materials is a challenging target. The cationic fluorescent nanogel thermometer, prepared with a new cationic radical initiator [6], can be taken up by mammalian cells. Sensitive and noncytotoxic fluorescent polymeric thermometers, based on the combination of a thermo-responsive polymer and a fluorophore sensitive to polarity and hydrogen bonding, are described in this article. D’Souza et al. compare the fluorescence properties of BODIPY fluorophores and report a closely related temperature-sensing system [7]. A sharp response to temperature variation was observed after wrapping one of the BODIPY derivatives in Pluronic copolymers consisting of hydrophilic poly(ethylene oxide) and hydrophobic poly(propylene oxide) units.

Ions are the universal targets of fluorescent sensors. As I mentioned at the beginning of the introduction, polymeric architectures can provide unprecedented sensitivity and selectivity. Three elegant examples are reported in this Special Issue. Kyhm et al. describe a sensing system for potassium ion (K^+^) in aqueous solution. This system combined the characteristics of fluorescent polyfluorene and a 15 base-aptamer that could pack K^+^ [8]. The aptamer was fluorometrically labeled with fluorescein and rhodamine. Upon binding K^+^, variable fluorescence outputs by the sensing system via Förster resonance energy transfer (FRET) were observed. Lin et al. synthesized novel silane-containing polythioethers that fluoresced in ethanol [9]. Selective quenching by ferric ions (Fe^3+^) was achieved by functionalizing the polythioether end groups with suitable sulfhydryl compounds. Preliminary experimental data obtained using human epithelial carcinoma (HeLa) cells were also reported. Seitz et al. prepared poly(*N*-isopropylacrylamide)-based nanoparticles that contained fluorescein and anilinodiacetic acid units and utilized them as sensors for cupric ions (Cu^2+^) in water [10]. While the nanoparticles intrinsically responded to changes in temperature, remarkable fluorescein quenching by Cu^2+^ was observed at a fixed temperature.

Molecular sensors require a special design approach that differs from that used for ion sensors, because the strong ionic interactions between the sensors and target ions and the coordination between ligands and target ions are absent. The oxygen sensing mechanism is unique, since quenching is due to collisions between molecular oxygen and the sensor. Peng et al. prepared ratiometric luminescent nanoparticles for oxygen sensing [11]. The nanoparticles consisted of polystyrene and polyacrylate block copolymers. They also contained oxygen-sensitive Ru^2+^ complexes and oxygen-insensitive Tb^3+^ complexes to afford a ratiometric emission signal. The polymeric structure around the complexes prevented interferences by other ions and molecules. The authors then demonstrated its application for sensing oxygen in model tumor spheroids. Zhao et al. developed a fluorescence sensor for adenosine triphosphate (ATP) in aqueous solution and HeLa cells using polythiophene that bore boronic acid and quaternary ammonium moieties [12]. The boronic acid and quaternary ammonium groups in the polymer bound to the diol and phosphate groups in ATP, respectively. ATP binding by polythiophene led to the formation of supramolecular aggregates, which quenched polythiophene emission. Tanaka et al. synthesized polyhedral oligomeric silsesquioxane that attached to Ru^2+^ complexes [13]. Electrochemiluminescence by the silsesquioxane was not significantly quenched by oxygen. In contrast, the water pollutant oxytetracycline markedly quenched silsesquioxane electrochemiluminescence after the oxidization of oxytetracycline at an electrode. Oxytetracycline sensing could thus be performed in a phosphate-buffered saline (PBS) without requiring a degassing procedure.

Molecular imprinting is an extraordinarily powerful polymer chemistry technique used to construct selective sensors. A polymeric receptor that is extremely specific to a target can be created via polymerization with the target molecule. Zhang et al. used this technique to prepare luminescent multilayered nanoparticles [14]. The cores of the nanoparticles contained iron oxides and luminescent quantum dots. The target molecule, bisphenol A (2,2-bis(4-hydroxyphenyl)propane), could be trapped in the 3-aminopropyltriethoxysilane-based shells. The nanoparticles were applied for the optical detection of bisphenol A in tap water and lake water. Bisphenol A bound to the nanoparticles quenched emission by the quantum dots in the cores in a linear fashion. The same technique can be extended beyond molecules to detect biological species. Wang et al. molecularly imprinted a polymer to detect the Gram-positive pathogenic bacterium, *Listeria monocytogenes* [15]. The polymer bound to *L. monocytogenes*, but it did not bind to *Escherichia coli*, *Staphylococcus aureus*, or *Salmonella*. *L. monocytogenes* was spiked into milk and pork, and detected through significant quenching of the luminescent polymer.

I am less familiar with the themes of the other contributions in this Special Issue, which make them more interesting to me. Matsumura and Iwai investigated variations in the microenvironment related to the complexation of poly(acrylic acid) and polyacrylamide using 9-(4-*N*,*N*-dimethylaminophenyl) phenanthrene, a polarity-sensitive fluorophore [16]. Complexation of the polymers was pH-dependent, and it was accompanied by significant changes in the local polarity. Warman et al. fabricated a poly(*t*-butyl acrylate) macrogel 24 mm in diameter, which contained fluorescent pyrene units to mimic the human eye [17]. The authors suggest that the poly(*t*-butyl acrylate) macrogel would be useful for controlling radiotherapy dosage, because the dose-related effects of the beam on the eye can be visualized in a three-dimensional fluorescence image of the macrogel. Ayesta et al. dissolved rhodamine B in methanol and inserted the fluorescent dye into poly(methyl methacrylate) optical fibers [18]. The authors showed that the penetration rate and fluorescence behavior of the rhodamine B-doped fibers were temperature-dependent. Their findings will be helpful for the development of new sensor materials. Timofeyev et al. coated fluorescent poly(allylamine hydrochloride)/poly(sodium 4-styrenesulfonate) nanocapsules with polyethylene glycol and monitored their circulation in a model amphipod, *Eulimnogammarus verrucosus* [19]. Knowledge about the distribution of the nanocapsules over time and their toxicity will hasten their application as sensors to monitor the physiological status of biological species.

Finally, I would like to express appreciation for the great editorial contributions of Liz Li and Zora Zhu at MDPI. I was kindly encouraged to work as a guest editor during a pleasant conversation with Ms. Hannah Guo, who attended a MDPI booth at the World Polymer Congress Macro2018 in Cairns, Australia. It is hoped that the information provided in this Special Issue will facilitate significant advances in polymer science in the future.
